# Medical Essays

**Published:** 1846-04

**Authors:** 


### Art. XI.

*Vermischte Abhandlungen aus dem Gebiete der Heilkunde,* von einer Gesellschaft practischer Aerzte zu St. Petersburg. Sechste Sammlung. —*St. Petersburg,* 1842.

*Medical Essays.* By a Society of Physicians in St. Petersburg. Volume the Sixth.—*St. Petersburg,* 1842. 8vo, pp. 396.

After a period of seven years another volume of Transactions has been published, by the Association of German Physicians, in St. Petersburg. The editors claim the indulgence of the profession for this long delay, on the plea that the printing of German works is encompassed with so many difficulties in the Russian capital, and, also, that unremitting professional labours left to the physician scarce any time for the scientific elucidation of his practice.

The volume before us is the sixth of the series, and without imparting anything very new or extraordinary in medicine, it may yet be considered valuable, as affording a fair criterion of the state of the healing art in St. Petersburg.

I. *Statistics and Treatment of Insanity at St. Petersburg;* by Dr. Herzog. This is an interesting account of the Asylum for the

Insane in the Russian metropolis. The treatment, and the general management of the unfortunate inmates, appear to be conducted on the most humane and scientific principles. From the ample tables given at the commencement it appears that here, as elsewhere, the bachelor is more liable to insanity than the married man; while, at the same time, the proportion of the insane is much greater in the higher and in the educated classes of society, than among shopkeepers and the artisans and labourers of a still lower grade. More than one half of the cases proved incurable. Of those who recovered, the great majority belonged to the lower classes, and complete returns to health were especially frequent among those individuals who, at an early period of their disorder, had disturbed the public peace and had been in consequence transferred to the asylum. The richer classes, of course, refrain as long as possible from placing their friends and relatives in such institutions, and continue to hope for a cure under imperfect treatment at home, till recovery becomes almost impossible. A full and lengthy account is given of the entire management of the institution, and we here extract a few lines as to the employment of the insane.

"The female lunatics are chiefly busied in the household duties, while the males, during the summer months, are busily occupied with the hay harvest, and in winter, when the severity of the season confines them to the house, they manufacture thousands of pill-boxes, and articles in pasteboard for the supply of the shops of the apothecaries. The more educated are occasionally employed in illuminating manuscripts, and some write to the dictation of others."

In the second portion of this paper, entitled, "Considerations on the treatment of the insane," Dr. Herzog develops his own views of mental disease, and we must allow that they are expressed with that candour and consideration for the opinions of others which is characteristic of a truly scientific mind. With most writers of the present day, Dr. Herzog denies that congestion or inflammation of the brain is necessarily present in insanity, and, if it should occur, he looks upon it rather as a complication and consequence of the malady than as its original cause. Nor does he allow of any close connexion between the suppression of hemorrhoidal or menstrual discharges and the outbreak of insanity. He enumerates the different causes of mental disease, as follows:

1. A certain hitherto unexplained condition of the nervous system characterized by great indifference to external agencies, as to heat, cold, &c. &c., and which cannot be traced to disorder of any particular organ. A similar state of the nervous system is often observed in constitutions worn out by drunkenness or by debauchery.

2. The brain, as the centre of the nervous system, must necessarily be looked upon as intimately connected with insanity, yet inflammations of this organ are not frequent at the commencement of mania, nor are we to regard the supervention of strong pulsations in the carotids, headache, brilliancy of the eyes, or sleeplessness, as indicating inflammatory action at a later period. But Dr. Herzog does not deny that secondary inflammation may arise in the brain, as a consequence of pathological changes which have taken place in that organ, through the influence of maniacal excitement. We must confess that the distinction here drawn is open to objection, and that we find here, as everywhere, the difficulty of defining the precise limits between irritation and inflammation.

3. Disorders of the digestive organs certainly exercise a very important influence on all cases of hypochondriasis and melancholia; the mental affection is constantly connected by the patients themselves with abdominal derangement, but no such relationship can be traced in other forms of insanity.

4. Diseases of the sexual system undoubtedly occasion certain varieties of mental disorder, and the most prominent of these is nymphomania. To this class also Dr. Herzog refers the melancholy cases arising from unrequited or restrained affection (Wahnsinn aus Liebe), which are but too frequent among females of the purest life and manners.

5. The sudden suppression of the lacteal secretion has occasionally a similar effect, though the disorder so well known as puerperal mania may occur quite independent of such causes. Dr. Herzog justly cautions us against interfering too vigorously with the numerous local maladies which may arise in the course of insanity, though he repeats that the cerebral irritation, so constantly accompanying the disease, may sooner or later occasion organic changes in the brain, and should, therefore, be combated by antiphlogistics. Still, he thinks, there is greater danger to be feared from excessive depletion than from over-caution in the use of the lancet, and the tact which must guide our hand in this respect can only be acquired by long experience.

The principal diseases which ensue as complications of insanity, are spasmodic and paralytic disorders, and derangement of the organs of digestion. Dr. Herzog insists strongly upon the danger of confining our practice to the routine treatment of these maladies, and affirms that those remedies alone will be successful which are directed against the original disease. In this assertion we need hardly say we cannot entirely concur, nor is it made so positively as altogether to exclude these secondary disorders from our consideration. In treating of the use of medicines in disorders of the mind, Dr. Herzog divides them into two classes, viz.:

A. Those which directly act upon the nervous system, as intoxicating and narcotic drugs, the employment of which, however, is greatly limited by the cerebral irritation which so often accompanies these maladies. Still in those cases of insanity, and they are by no means so rare as has been asserted, where no cerebral irritation exists, opium in the hands of medical men, and alcoholic liquors administered by empirics, have been found of the greatest benefit. This latter mode of treatment the author states to have been far too much neglected by the profession. The evil consequences that may have occasionally ensued from its employment, would rather indicate that it requires to be directed by a skilful hand than to be entirely thrown aside.

B. The second class of medicines are those which act directly upon the intestinal canal, or upon the external skin, as purgatives, emetics, and warm baths. Our author has seen but little benefit derived from counter-irritants, as issues, or the tartar emetic ointment, although the frequent outbreak of the malady on the suppression of accustomed discharges would seem to indicate their employment.

To direct our external means so as to influence the disordered *mind* of the patients is then the chief indication in mental disease. We must endeavour, in the first place, to act upon their feelings and sensibilities, without addressing ourselves solely to their understandings; but at the same time it should be remembered that the moral treatment is not always to be directed according to the former character of the individual, as this may, and usually does, undergo a total change at the very commencement of the malady. The habits, mental and physical, of early life continue, however, for a long period in the insane, and it is by means of these that we can hope to obtain the greatest influence over the patient. The agencies and circumstances which attract the attention, without unduly exciting the mind of the insane, are the only ones which should be employed in mental disease, where everything should be avoided which might confuse or give a shock to their reasoning or moral faculties.

In certain cases our author has found it necessary to separate some patients from others, and in rarer instances he has had recourse to complete isolation—a mode of treatment, however, which can with difficulty be completely carried out in large lunatic asylums.

All harsh language or reproaches towards patients are useless, if not injurious; and yet, as Dr. Herzog very justly observes, quite as much injury may be done by the opposite extreme of excessive tenderness and sentimentality, which is equally if not more foreign to the altered feelings and sensibilities of the patient.

To attempt a cure by reasoning with a patient upon the folly of his actions, or by opposing in strenuous argument the fixed ideas which constitute his delusion, will only aggravate the disorder. Dr. Herzog here adds some excellent observations upon the degrees in which we should yield to the patient's wishes and desires for luxuries, solitude, study, or amusements. Much here depends upon his previous habits, as also upon the question whether these desires are the mere offspring of a disordered imagination, or the consequence of the previous circumstances of the individual. "A maniac who when in health was accustomed to walk alone, may justly be allowed to pursue the same course when diseased; nor should we consider it as a favorable symptom if he were willingly to join the crowd of patients in their daily promenades." The author warns us carefully against forcing work, manual or mental, on the insane when they exhibit no disposition thereto; and he believes that employment is chiefly profitable when they begin to show symptoms of returning health.

*Recreation.* In the asylum at St. Petersburg rich and poor are, by a government ordinance, treated alike; and this produces the most injurious consequences on the wealthier classes of the insane. These ought to be surrounded as much as possible with all the comforts and conveniences of their homes; and Dr. Herzog calls earnestly for an amelioration which has already been partially effected in this country and on the continent, viz., to exclude as much as possible all semblance of confinement within the walls of a prison, such as our lunatic asylums formerly were. Music and painting have been much extolled as recreations in mental disorders; but it is curious that they have never produced any beneficial effects—at least this is asserted by Dr. Herzog; and we may perhaps ascribe a good deal of this bad success to the over-excitement produced by such pursuits, and also from a greater degree of freedom being required for their due cultivation and influencing of the mind, than can be allowed in a large public institution.

The author next discusses the long agitated question of the advantage of change of scene and of position to insane patients; and he concludes that for restless and irritable individuals no alteration should be made, while in the milder description of cases it is decidedly beneficial.

Of religious exercises our author speaks very cautiously. No good, he thinks, can result from forcing religious contemplations or prayers upon the insane; but the favorable impression produced upon some by the solemn ceremonies of the Russian and Catholic churches is unquestionable. Of all modes of treating an insane patient, that of placing them in a private establishment of their own in the country is to be preferred; but from the great expense this necessarily entails, such an advantage can fall to the lot of a very few. The very fact of being consigned to an asylum must always be injurious to those who have any feelings of dignity or self-respect remaining. In many well-conducted asylums considerable attention is now paid to separating the slightly affected or the convalescent from the furious maniac or the idiot; but much yet remains to be accomplished before this most necessary separation can be completed in a satisfactory manner. Dr. Herzog suggests that to each asylum there should be attached a house of recovery, to which convalescents could be transferred; and as the number of those likely to recover, according to all writers, cannot be carried higher than the fourth or fifth part of the inmates of asylums, so the additional expense and maintenance of such a house of recovery would be proportionately small.

Our analysis of Dr. Herzog's essay, which occupies eighty pages of the original volume, has been somewhat extended; but we offer no apology to our readers for laying before them the results of many years of careful observation, by one eminently well calculated for the management of the insane. Dr. Herzog's essay exhibits a sound judgment, and a healthy moral feeling runs through the whole, which assures us that the amelioration he suggests will be beneficial to this, the most unfortunate, class of our fellow-creatures.

II. *Cases of Insanity from Wounds of the Head;* by Dr. Herzog. These seven cases are in themselves by no means curious, being all merely slight sabre-cuts or gun-shot wounds of the face or head; and in one case there was a slight fracture of the cranium, while in the last instance the disease ensued after the operation for strabismus. Four of the patients died, and in three an examination of the head was obtained, but *no morbid appearances could be discovered.* From these somewhat imperfect data Dr. Herzog has drawn some singular conclusions. "Medical men in general," says he, "would assert that the blows and contusions received by these individuals, though not severe enough to cause immediate violent action on the nervous system, yet might produce that commotion of the cerebral functions, which, though suppressed for a while by the vis medicatrix naturæ, would sooner or later manifest its destructive ravages." Against this opinion Dr. Herzog urges the following arguments: 1st. That no organic lesion, as a consequence of the injuries received, months or years before, could be discovered on dissection. 2. That in three or four of the cases no possible injury or concussion of the brain could have taken place, [?] as the wounds were not of a nature to effect this, and yet, nevertheless, insanity ensued.

To this partial reasoning we object that most of the cases occurred in old or invalided soldiers, who, from the want of employment and from their idle and dissipated habits, are peculiarly liable to insanity. Nor does it naturally follow that the mental disease would not have been developed in these individuals without any previous injury having occurred. Moreover, six out of seven of the patients above referred to had been wounded in the head; and though the majority were merely flesh wounds, still the nervous centres may have been more contused and disturbed than we are aware of. In all the seven cases convulsions and symptoms of paralysis occurred at an earlier or later period, along with the mental affection. Dr. Herzog then asks, "May not superficial injuries of the head sooner or later induce serious alterations in the nervous system? and may not the germ of the malady be formed in the wound, and after a long period of time develop itself in connexion with the nervous system?" Dr. Herzog asserts that insanity rarely occurs after *very severe* wounds of the head. In short, Dr. Herzog would make us believe that slight injuries of the cranium and of the nervous centres may, as in the case of the bite of a rabid animal, leave in the wound an undeveloped germ of future disease, from which at a later period may arise insanity, as in the other case hydrophobia. We cannot coincide entirely with his views, but the occurrence of mental disease after slight wounds is well worthy of the investigation of pathologists. It would be interesting to ascertain exactly the proportion of individuals thus secondarily affected. We believe that they would be found very few in number, and not enough to alter our suspicion that insanity might have occurred in the cases before us, had no wounds at all been received.

III. This article contains details of five *cases of sudden death*, by the veteran Professor Busch. It is, however, manifestly imperfect, as no post-mortem examinations of the cases are recorded; nor is it mentioned that they were sought for. We leave them, therefore, unnoticed.

IV. This article is from the same author, and contains details and observations on two remarkable cases which terminated fatally. We must, however, here, as well as in the previous instance, regret the scantiness of pathological details; for, in the first place, the head alone was opened, and in the second no post-mortem examination was made at all, though, from the details of the symptoms in both instances, the stomach was at least as probably the seat of disease as the cerebral system. Both patients were young females of the higher classes, and in each the approach of the menstrual period, though the discharge had never yet appeared, was indicated by the usual premonitory signs. The patients had retired to rest in good health, and awoke about 3 a.m. with vomiting, which after continuing incessantly for some hours, was succeeded by furious delirium. The usual revulsives and counter-irritants were employed, and the symptoms of excitement subsided, but were succeeded by a state of quiescence, which was occasionally interrupted by general convulsions until death occurred on the third day. Slight congestion of the blood-vessels of the brain was found upon dissection. In some respects these attacks resembled the epidemic cholera, but many of the characteristic symptoms, such as the rice-water evacuations, and the blue, pinched appearance of the countenance and of the extremities, were wanting here. Professor Busch's conclusions, drawn from the symptoms alone, are by no means satisfactory, and he believes that life was extinguished by the vital energies of the ganglionic and cerebral system becoming totally exhausted from repeated vomiting. The cause of the malady he does not however satisfactorily elucidate.

V. and VI. These two articles are also by the same author, but are devoid of interest. The former is that of a brother and sister whose maladies were almost identical, and in the latter we find the details of a case of insanity, which appears to have resulted from a venereal affection.

VII. *Report of the Imperial Foundling Hospital of St. Petersburg, from* 1834 *to* 1840; by Dr. Philipp Doepp. Much improvement has recently been effected in the condition of the foundlings of the Russian capital. The new Lazareth, as it is termed, for the older female infants contains 160 beds, and is a building of two stories in height. A similar edifice has recently been erected for male foundlings, and the utmost care has been taken to render this great institution in every way serviceable to the end for which it was designed. The organization of this hospital seems to be nowise inferior to its external appearance. The beds of the foundlings and their nurses are all placed against the transverse or partition walls of the wards, so as to avoid all danger of cold, or currents of air from the neigbourhood of the windows. So ample and complete are its accommodations that many parents have sought and obtained admission for children born in wedlock, thus permitting their legitimate offspring to be stamped with the brand of bastardy, for the selfish end of procuring them an excellent gratuitous education. By this abuse the number of foundlings became alarmingly increased, till an ukase of the Emperor, bearing date of the 25th of June, 1837, ordained that all children reared in the foundling hospital should, on attaining the requisite age, be apprenticed to artisans or to peasants, or if they themselves particularly desired it, should be enlisted as common soldiers. The effect of this prudent though somewhat harsh measure, was that 860 children were withdrawn by their parents, and 324 others were taken away during the succeeding years. By a further order of the 15th of May, 1839, the custom of delivering a ticket of reception (Empfangschein) to the parents was abolished, so that from 1840 the institution has been restricted to its original intention, viz., that of affording an asylum to illegitimate children *really* deserted by their parents. This latter ordinance has diminished the number of foundlings by one fifth.

Dr. Doepp has found that the observations upon diseases occurring in this institution, and which were published in the preceding volume of these transactions, have been fully corroborated by his seven years' additional experience.

1. Diseases among the older children of the foundling hospital:

*Morbilli sine catarrho, or false measles.* An epidemic of this kind was observed in 1836 and again in 1839. The eruption appeared exactly like the regular disease, but without any febrile or catarrhal symptoms, and it vanished from the surface in about four days. Above 100 children were thus affected in the institution, and the epidemic was observed also in many cases in the capital. Many instances of this kind also occurred during the great epidemic of genuine measles in 1839, when many children were attacked with the regular disease, who had previously suffered from the non-catarrhal form above referred to.

*Genuine measles.* This epidemic was one of the most severe and malignant that Dr. Doepp had ever witnessed. The symptoms were at first inflammatory, and became subsequently nervous. Of the children in the institution, 202 were attacked, but only three died, one of *phthisis florida,* and the other two of pericarditis. The peculiar odour, resembling that of goose feathers, first noticed by Heim, was not detected in these cases, even though so many affected individuals were congregated in one apartment. Belladonna and sulphur were found perfectly inert as preservatives.

*Scarlatina.* Only *two* cases have occurred in the foundling hospital during the last seven years, and one of these proved fatal. Dr. Doepp cannot well account for the freedom of the institution from this dangerous malady, during a period when it has been repeatedly epidemic and extraordinarily fatal in the city of St. Petersburg.

*Scrofula.* Dr. Doepp has latterly used, with very excellent results, the cod-liver oil in scrofulous affections of even very young children. Nor was he deterred from its employment by diarrhea, or mesenteric disease: in both cases a favorable termination generally ensued. He is also a firm advocate of iodine, but insists much upon combining its employment with free exercise in the open air.

The mortality in this division of the institution is extremely small, scarcely exceeding one per cent. during the last six years. Tubercular phthisis and chronic diseases of the intestines were the chief disorders that terminated fatally.

2. Diseases occurring in new-born infants:

*Ophthalmia neonatorum.* Dr. Doepp does not believe a bright and intense light to be a cause of this disease, as those children that lay nearest to the windows were not more affected than others. It never became epidemic in the hospital, and was certainly, in most cases, occasioned by cold.

*Erysipelas neonatorum.* This is still a frequent disease, especially after vaccination. Dr. Doepp states expressly that he has found erysipelas most frequent in infants when they had been vaccinated with lymph taken from a highly inflamed pustule or vesicle. And he has been able to affirm this with greater certainty from the results of the practice he has long followed, of vaccinating each child in both arms, but with different lymph, and he constantly produced erysipelas in that arm which had been vaccinated with lymph from a vesicle highly inflamed. The erysipelas was best cured by free and repeated scarification.

The epidermis, or its succeeding scab, is occasionally torn off from the pustules of vaccination; and the latter are then very prone to change into erysipelatous ulcers, from whence there flows a quantity of an acrid secretion. Dr. Doepp's practice, in such cases, is to fill the excavation with finely pulverized flores zinci, which forms an artificial crust, and can be easily renewed by the same process, if torn off before the part beneath has become completely cicatrized. But at times these ulcerations will not heal at all, and continue obstinately to spread, when we should probably be justified in considering them of a syphilitic origin, and that more unequivocal marks of that disease will speedily appear.

*Syphilis neonatorum.* Richter, Henke, Jörg, and others have denied the existence of this disease, but the experience of Dr. Doepp has convinced him that it is by no means uncommon. Several cases of true syphilitic eruption appeared in various infants very soon after birth, and yet the mothers, to all appearance, were quite free from any venereal taint. In such cases Dr. Doepp suspects, with Dr. Evanson and many other writers, that the infection has been derived from the male parent. Small doses of mercury were most useful in effecting a cure, but no benefit was obtained thereby if the child was taken from the nurse's breast. Sooner or later the nurses themselves became affected with syphilitic sores and eruptions, which required for their cure a vigorous course of corrosive sublimate. The non-mercurial plan of treatment totally failed.

*Cephalæmatoma neonatorum.* In a private practice extending over the long period of twenty-six years, Dr. Doepp has observed this remarkable affection only thrice, while in the foundling hospital, during the space of eleven years, it has occurred in not less than 262 cases, and in all, excepting two, the parietal bones, near their superior portion, were the seat of the disease. The average amount of cases was 1 in 190 of the whole children in the hospital: 11 post-mortem examinations were made, including 8 who died of other disorders when thus affected. The results of the dissections were the following:

*a.* In none of the 11 cases was any perforation of the skull observed, nor any effusion of blood upon the inner surface thereof.

*b.* All the 262 patients were brought to the hospital with the affection already existing, though many were not above a few hours old. But the tumour was often observed to increase in size after the arrival of the children at the hospital, at least during the first three days, after which no further enlargement took place, except in those cases where the tumour having been opened had again become distended.

*c.* It was only in nine cases of the whole number that Dr. Doepp could ascertain the nature of the labour. All the nine had been easy and every way natural, and the mothers were strong, healthy, and not overburdened with fat. Three were primiparæ; the previous children of the other eight had been perfectly free from any signs of this affection.

*d.* The cephalæmatoma seemed to be always beneath and separate from the scalp; it was a *tense* fluctuating tumour; the integuments were of normal colour, and could, even in the most distended condition of the swelling, be freely moved backwards and forwards over its surface. The pulsation noticed by Naegele and others was never observed, nor did pressure on the tumour seem to cause pain to the infant. Its form was usually oval, but was sometimes altered by two or three tumours being placed in close apposition. The largest tumour that was observed in the hospital measured about four inches by three, and several were seen not larger than a hazel nut.

*e.* The hard ring around the tumour was never wanting, though at an early period of the affection it was less observable than afterwards. It was *most distinct* whenever the tumour was emptied by incision, and gradually disappeared then in about five days' time. Dr. Doepp carefully dissected two cases for the express purpose of ascertaining the true nature of this callous ring. After removing the external integuments, he found that the tumour was formed by the pericranium distended with blood. Upon incising the swelling and washing out the contents, he observed the callous ring to consist *solely* of a layer of coagulated blood, which formed all round at the point where the pericranium had been upraised from the bone. By a continuous stream of water this layer could be dissolved away, and with it the callous ring entirely disappeared. The pericranium was everywhere healthy, as was also the bone beneath. No appearances of inflammation could be detected in either.

Dr. Doepp does not, however, deny that, in certain cases when the tumour has been opened, and much suppuration has ensued, the bone may become carious, and thereby the exterior table of the skull with the diplöe may be destroyed. In such instances the circumference of the carious portion would necessarily present a sharp unyielding edge. In one skull in Dr. Doepp's possession the absorption of the diplöe appears to have commenced first, and the external table over the absorbed portion was depressed in the centre, but elevated at its circumference, so as to form a callous ring of true bone. The termination of this affection was almost always favorable. Two children died out of 265, and one of these from violent phrenitis, perhaps occasioned by cauterizing the interior of the tumour with nitrate of silver. The cause of cephalæmatoma Dr. Doepp confesses himself unable to explain, and he is quite dissatisfied with all theories hitherto promulgated.

The treatment appears to have been simple and sensible. Fomentations were applied at first, but if no disposition to absorption manifested itself before the end of the second week the tumour was opened by a small incision, four or five lines in length, and the extravasated blood was pressed out. The fear of hemorrhage, so dangerous to a new-born weakly child, deterred Dr. Doepp from emptying the tumour at an earlier period, and he recommends that the operation, on the other hand, should not be delayed beyond the fortnight, as absorption of the bone will then probably commence.

*Hermaphrodism.* In the list of deformities observed in the institution, a singular case of female hermaphrodism is related. The clitoris was above an inch (English) in length, and possessed a perfect glans and præputium. From its interior surface a groove extended to a small opening in the perineum. The integuments hung down on either side of this orifice, exactly like a divided scrotum into which the testicles had not as yet descended. No urine flowed from the above-named orifice in the perineum, but still it might have been considered justly as the orifice of the urethra, as the child was born with a hernia of the completely retroverted bladder. An inflammatory attack in the bowels carried off the infant at the age of ten months, and upon dissection a double vagina and a double uterus were discovered. The other internal female organs presented nothing abnormal.

*Mortality.* The deaths in the institution continue at the rate of 50 per cent., annually. The annual mortality in the excellent Foundling Hospital at Vienna is not less than 66½ per cent., a superiority of mortality which Dr. Doepp attributes to the children in the latter establishment being almost entirely brought up by hand. More than once has this plan, on account of the enormous expense of wet-nurses, been proposed in the hospital of St. Petersburg; but, were it adopted, Dr. Herzog says not one twentieth of the children would be saved.

VIII. *History of an operation for varicose aneurism*; by Dr. Doepp. The child, a female, was two years and two months old, and the tumour occupied the right cheek, being in size equal to the clenched fist of a man. The operation, which Dr. Doepp considers to be new, consisted in transfixing the tumour with a needle four inches long, to which was attached a firm double ligature. The needle was carried through the tumour and the ligature then firmly tied over a hand compress. Suppuration soon came on, and a perfect cure ensued without any bad symptoms. Our readers know that this operation is not new; it has been frequently, we believe, performed both in this country and abroad.

IX, X, XI. These articles are, respectively, cases of *encysted tumour of the brain,* of *heart disease,* and of *abdominal emphysema.* The first is related by Dr. Doepp, but as its details throw no additional light on the pathology of the cerebral system they may be passed over. The second case, by Professor Lichtenstädt, is merely one of hypertrophy of the heart, with ossification of its valves. The third, by the same author, is interesting only in so far as the abdominal emphysema occurred as a complication of erysipelas of the face, and the distension of the intestines was apparently occasioned in a great measure by occlusion of the ilio-cæcal valve from a melanotic tumour situated in that portion of the intestine. The cæcum was found deeply ulcerated, and there was also a stricture of the sigmoid flexure of the colon.

XII. This is a short paper by Dr. Wolff on *transfusion,* which contains nothing interesting.

XIII. *Report of the Children's Hospital in St. Petersburg*; by Dr. Weisse. This establishment was founded in 1834, and consisted then of only 60 beds. It now affords accommodation for 102 children. The report embraces a period of six years, from the foundation of the hospital to January 1841. The internal arrangement of the building and its domestic economy are given at great length, but we have only room for the second part of the report, wherein is given a history of the more remarkable cases of disease that have occurred there during the above-mentioned period.

*Gangrene of the genitals.* Two cases of this affection in the female organs have been observed. One of these gave rise to an accusation of rape against an individual of the higher rank in St. Petersburg; the disease, indeed, seemed to have been quite unknown to the practitioners in that capital, till their attention was called to its existence by Dr. Weisse, who, however, candidly acknowledges that he himself only became acquainted with the disorder by casually observing in Horn's Archiv, a translation of Isuard Cevoule's Essay on Gangrene of the Labia in female children. Dr. Weisse considers this malady to be identical with noma, and proposes to designate it *noma genitalium.* It has occasionally, he says, been observed along with that disease in the same individual.

*Measles.* Dr. Weisse in five or six cases observed the eruption of measles to supervene on the convalescence from typhoid fever. When patients labouring under scabies were attacked with measles the former malady disappeared, during the prevalence of the exanthema, but returned in full force after the latter had passed away.

*Ascites.* In three cases Dr. Weisse has tried parcentesis, but without any permanent benefit; indeed, in one case, death was directly accelerated by the operation.

*Caries of the frontal bone.* This arose from a slight but neglected wound over the right parietal bone, from which there gradually ensued an enormous suppuration beneath the scalp. Many pounds of pus were pressed out through the original very minute wound on the first day that the child was seen by Dr. Weisse. The frontal bone was carious to a considerable extent, and an immense discharge flowed for some time from the wound, but the child eventually recovered, after remaining four months and a half in the hospital.

*Hydrocephalus acutus.* Dr. Weisse, in an almost hopeless case of this disease, made trial of corrosive sublimate internally, as recommended by Dr. Spiritus, in Hufeland's Journal. A twelfth of a grain was given every two hours in distilled water. The patient, a girl of five years of age, eventually recovered, but many small abscesses appeared during convalescence beneath and in the scalp, and which occasioned much pain, though they probably acted beneficially as counter-irritants. Of course not a shadow of proof exists that the disease was cured by the mercury. The reverse is much more probable.

*Bifurcation of the aorta.* A boy of fourteen died with the usual symptoms of heart disease, and acute pericarditis was discovered on dissection. The aorta shortly after its origin from the left ventricle divided into two branches, which after a short course again united, forming a dilated sac at the point of reunion. The left carotid and subclavian sprang from the left branch of the aorta, from the right branch there arose only the right subclavian, the right carotid springing from the divarication of the aorta.

*Permanent contraction of the limbs in children.* Dr. Weisse has observed several instances of this disease, but does not add much to what has been already written on the malady by Guersent and by Baudelocque. Dr. Weisse does not believe the symptoms to arise solely from cerebral disease, but he has more frequently observed it as a consequence of the irritation of worms, of masturbation, or of the approach of the menses in young girls.

*Exanthematous diseases.* In the space of four years Dr. Weisse has observed sixty-one cases in which an acute exanthema has supervened upon some other cutaneous disorder, or upon another disease. In forty-two of these the supervening exanthema was measles; in eight, scarlet fever; in six variola; in three varioloid; and in two varicella. Five of the patients suffered, each in quick succession, from three different exanthemata. The comparative rarity of smallpox after other diseases is easily explained by the influence of vaccination, but against scarlatina no such protecting influence is known to exist. Dr. Weisse suggests that, as no case is known (?) where scarlatina has supervened upon true variola, the tendency to the former may be destroyed by genuine smallpox, and thus perhaps may be explained the great increase of virulence in the scarlatina of later years. "Most practitioners of the present day," observes Dr. Weisse, "will admit that our scarlatina is no longer the smooth red blush formerly described, but is on the contrary almost always the miliary form of the disease." With Dr. Jahn, of Meiningen, he considers the vesicles of our present scarlet fever as the residuum of the tendency to smallpox (pocken-anlage), existing in the constitution. Dr. Weisse has even seen some of the vesicles surrounded by a distinct areola, and filled with a fluid resembling pus.

The acute exanthemata are more easily communicated to those labouring under chronic than under acute disorders. Patients suffering from fever, are not liable to the exanthemata until their disease is subsiding.

*The mortality* in the children's hospital has of late years been only 14 per cent., while in similar institutions in Berlin, Paris, and Vienna, the deaths have amounted to 24 per cent. annually. Into all these other hospitals, however, it must be remembered that children merely labouring under scabies are not admitted.

XIV. *On the uses of calendula and of fuligo splendens in the diseases of women*; by Dr. Ockel. Both these remedies, the plant and the root, were employed by ancient physicians. Dr. Ockel administers the extract of calendula in a certain condition of the uterus, much resembling that described by Professor Simpson and others as hypertrophy of that organ. The uterus descends low down into the pelvis and is much increased in size, thickened and indurated, though still to a certain degree elastic, and possessing none of the hardness of scirrhus. The uterus thus enlarged fills the entire pelvis, and the patient suffers from constant dragging pain, while the os uteri becomes extremely tender to the touch. The bowels in this disease are almost always much constipated, and require free evacuation by the usual purgatives, after which the ext. calendulæ should be given, with glauber salts three times a day. The cure occupies generally the space of five or six weeks. Dr. Ockel has occasionally observed this condition of the uterus to be connected with violent metrorrhagia, and in such instances he believes the calendula to be the only efficient remedy.

Fuligo splendens is extolled by Dr. Ockel as an excellent remedy against abortion.

XV. This is a history of *a case of chorea*, treated and cured (?) by the muriate of tin, in the dose one sixteenth of a grain for some weeks, by Dr. Person.

XVI. ***Report of the Private Ophthalmic Institution of St. Petersburg, from May,*** 1833 ***to May,*** 1841 ; by Dr. W. Lerche. Four cases of confirmed cataract were treated by galvanism, or rather by galvano-puncture. Three of the patients completely recovered their sight, but the vision of the fourth was little, if at all, improved. The history of these cases has been already published in several of the continental journals, and also in our own.

The volume concludes with a number of chirurgical cases by Dr. Salomon : none of these, however, are of any great interest. Dr. Salomon is evidently a pains-taking and prudent surgeon, and seeks rather to enlighten his brethren by recording his experience, than to astonish the medical world by the boldness and success of his operations.

As an appendix, we have the address presented to Professor Busch upon his fifty years' jubilee as a practitioner. In this address is contained an excellent recapitulation of the transactions of the German Medical Association, from its origin until the present period; the noblest tribute that could be paid to one who may justly be regarded as the founder of that Society.

A few typographical errors occur in this volume; but upon the whole it is a fair and good specimen of Russian typography. We sincerely hope that seven more years may not elapse ere we are favoured with another volume from our northern friends.

---

